# Harnessing the anticancer potential of *Moringa oleifera*: a safer, multi-targeted adjunct

**DOI:** 10.1097/MS9.0000000000004227

**Published:** 2025-11-06

**Authors:** Faseeha Abro, Nikil Kumar, Nitasha, Sinnha Kumari, Shanza Qazi, Muhammad Saad Khan, Aminath Waafira

**Affiliations:** a Department of Medicine, People University of Medical and Health Sciences, Nawabshah, Pakistan; b Department of Medicine, Liaquat University of Medical And Health Sciences (LUMHS), Jamshoro, Pakistan; c Department of Medicine, Jinnah Sindh Medical University, Karachi, Pakistan; d School of Medicine, The Maldives National University, Malé, Maldives

**Keywords:** anticancer, apoptosis, integrative oncology, Moringa oleifera, phytochemicals

## Abstract

*Moringa oleifera*, a nutrient-rich medicinal plant, demonstrates promising anticancer potential across various malignancies, including breast, colorectal, liver, lung, prostate, and oral cancers. Its bioactive phytochemicals – such as isothiocyanates, flavonoids, phenolic acids, and alkaloids – exhibit antioxidant, anti-inflammatory, apoptotic, and antiproliferative effects. Mechanistically, *M. oleifera* induces apoptosis by modulating *Bcl-2/Bax* expression, activating caspases, and generating reactive oxygen species (ROS). Its extracts and silver nanoparticles suppress metastasis-related genes and downregulate key oncogenes like *c-Myc* and *FSHR*. Molecular docking and network pharmacology confirm the affinity of its phytochemicals to cancer-regulatory proteins. Preclinical and limited human studies reveal a favorable safety profile with minimal toxicity at oral doses. *Moringa*’s diverse mechanisms, broad-spectrum activity, and low toxicity underscore its potential as an adjunct in cancer therapy. Future research should focus on clinical validation, standardized formulations, delivery systems, understudied cancers, and combinatorial regimens with conventional therapies to advance its role in integrative oncology.

*Dear Editor,*

Cancers such as hormone-driven breast cancer, colorectal cancer with Wnt and p53 mutations, liver cancer associated with viral infections, lung cancer associated with toxins and EGFR/KRAS mutations, androgen-sensitive prostate cancer, and invasive oral squamous cell carcinoma are all characterized by unchecked cell growth. The necessity of multi-targeted therapies has been proven by these molecular variations. While traditional treatments like radiation, chemotherapy, and surgery frequently have major side effects, medicinal plants and phytochemicals present safer, more beneficial options that may be able to fight cancer and improve patient tolerance[1].

## Phytochemicals of *Moringa oleifera* and their mechanisms

*Moringa oleifera* (MO), rich in bioactive compounds, shows strong antioxidant, anti-inflammatory, and anticancer properties through its phytochemicals like isothiocyanates, flavonoids, phenolic acids, alkaloids, and glucosinolates derived from its leaves, seeds, fruits, and roots^[1,2]^.

**HIGHLIGHTS**

- *Moringa oleifera* demonstrates *broad-spectrum anticancer effects* through apoptosis induction, anti-inflammatory actions, and antioxidant activity.
- Its bioactive compounds – *isothiocyanates, flavonoids, alkaloids, and phenolic acids* – modulate key oncogenic pathways such as *Bcl-2/Bax, c-Myc*, and *FSHR*.
- Molecular docking and network pharmacology studies support *multi-target interactions* of *Moringa* phytochemicals with cancer-related proteins.
- Preclinical and limited human studies indicate a *favorable safety profile* even at high oral doses with minimal adverse effects.
- Future research should emphasize standardized formulations, clinical trials, and integrative approaches, aligned with transparent reporting frameworks like the TITAN 2025 guideline.

Through apoptotic and antiproliferative mechanisms, MO shows anticancer benefits. The lectin cMoL, which is produced from seeds, increases mitochondrial ROS by activating caspases-3, -8, and -9. By upregulating *Bax* and downregulating *Bcl-2*, MO extracts control apoptosis. While MO-derived silver nanoparticles decrease colon cancer cell viability and repress genes linked to metastasis, MO root extract suppresses *FSHR* and *c-Myc* in ovarian cancer[1]. Furthermore, although the precise anti-angiogenic routes are still being investigated, MO phytochemicals may suppress angiogenesis and halt the cell cycle[2].

## Preclinical evidence: promise and potential

By inhibiting the growth of colorectal cancer cells and reducing the size and number of breast tumors, MO’s flavonoids and isothiocyanates have anticancer effects on a variety of cancer types[3]. Quercetin and kaempferol are examples of phytochemicals that control cell cycle, trigger apoptosis, and regulate proliferation^[1,2]^. Leaf, seed, and oil extracts exhibit cytotoxicity and anti-proliferative properties in oral squamous cell carcinoma, whereas nanoparticles decrease the production of TNF-α, HSP27, and IL-102. Evidence from Kambuno *et al’s in vitro* studies on cancer cell lines through apoptosis, ROS production, and cell cycle arrest pathways has shown that *Moringa* extracts exhibit cytotoxic effects on liver, lung, and prostate malignancies (Table 1)[1].

**Table 1:** Summary of preclinical anticancer effects of *Moringa oleifera* extracts and phytochemical

Cancer type	Active component/extract	Key mechanism of action	Evidence level	Reference(s)
Breast cancer	Leaf extract, benzyl isothiocyanate	Induces apoptosis, reduces tumor weight, modulates *Bcl*−2/*BaxBcl-2*/*Bax*Bcl−2/*Bax* ratio.	Animal study (*in vivo*), cell culture (*in vitro*)	Rojas-Armas *et al* ^[3]^
Colorectal cancer	Quercetin, kaempferol	Regulates cell proliferation (cell cycle arrest), induces apoptosis via caspase activation.	Cell culture (*in vitro*)	Kambuno *et al* ^[1]^
Oral squamous cell	Leaf nanoparticles	Cytotoxic, anti-proliferative, reduces expression of TNF−α, HSP27, IL−10TNF-\alpha, HSP27, IL-10TNF−α, HSP27, IL−10.	Cell culture (*in vitro*)	Budhy *et al* ^[2]^
Liver cancer	Seed/leaf extract	Induces apoptosis, G2/M cell cycle arrest, ROS generation.	Cell culture (*in vitro*)	Kambuno *et al* ^[1]^
Lung and prostate	Various extracts	General cytotoxic effects via apoptosis and cell cycle arrest.	Cell culture (*in vitro*)	Kambuno *et al* ^[1]^

## Safety profile in early human studies

Molecular docking and network pharmacology demonstrate that MO targets cancer pathways, indicating that its phytochemicals bind proteins that cause cell arrest and apoptosis[4]. While efficacy data remain preclinical, initial human studies like Stohs *et al*’s, which focused on safety along with its anticancer potential, have demonstrated an excellent profile[5]. No negative effects were observed in healthy persons who took 8 grams of powdered *Moringa* leaf every day for 40 days[5]. In an another trial, dosages up to 7.2 g/day for 7 days were associated with very mild, transient stomach discomfort[6]. Studies on the toxicity of several MO extracts showed no negative side effects at oral dosages of up to 2000 mg/kg. If proven in humans, *Moringa* could potentially offer safer, more tolerable medicines, with preliminary data indicating short-term safety[4].

## Safety, limitations, and future directions

MO has emerged as a promising source of bio-active compounds with broad therapeutic applications[7]. However, the existing evidence for its anticancer benefits is primarily based on preclinical studies in cell cultures and animal models, with a noteworthy lack of rigorous randomized controlled trials to prove therapeutic efficacy in humans. Future research should prioritize well-designed clinical trials, standardized extraction procedures, and investigations into molecular pathways, understudied malignancies, and synergistic effects with conventional therapies[8]. These endeavors will not only assist in the confirmation of efficacy but also provide patients and healthcare providers with the necessary evidence to make informed, confident decisions. As the interest in phytotherapeutics expands, it is equally crucial that future research on MO follows standardized, transparent reporting methods to enhance predictability and scientific rigor, as specified in the TITAN 2025 guidelines (Table 2)[9].

**Table 2:** Recommendations for future research on *Moringa oleifera*, guided by the TITAN 2025 Principles for Transparent Reporting

Research area	Key objective	Relevance to TITAN guideline principles
Standardization	Develop standardized extracts with defined phytochemical profiles, doses, and plant parts.	Enhances *reproducibility* and allows for meaningful comparison across studies.
Clinical validation	Conduct well-designed Randomized Controlled Trials in human patients.	Moves evidence from preclinical to clinical, assessing true *efficacy and safety*.
Mechanistic insight	Use omics technologies (genomics, proteomics) to clarify multi-target mechanisms.	Provides *transparent and detailed* understanding of molecular pathways.
Combinatorial therapy	Investigate synergy with conventional chemotherapy/radiotherapy.	Addresses *complex interventions* and potential for integrative oncology.
Safety and toxicology	Conduct long-term toxicology studies at various doses.	Ensures *ethical and rigorous* reporting of potential adverse effects.

## Data Availability

Not applicable.

## Research registration unique identifying number (UIN)

1. Name of the registry: N/A. 2. Unique identifying number or registration ID: N/A. 3. Hyperlink to your specific registration (must be publicly accessible and will be checked): N/A.
